# What Makes Adventitious Roots?

**DOI:** 10.3390/plants8070240

**Published:** 2019-07-22

**Authors:** Mathieu Gonin, Véronique Bergougnoux, Thu D. Nguyen, Pascal Gantet, Antony Champion

**Affiliations:** 1 Université de Montpellier, IRD, UMR DIADE, 34394 Montpellier, France; 2 Department of Molecular Biology, Centre of the Region Haná for Biotechnological and Agricultural Research, Palacký University Olomouc, Šlechtitelů 27, 783 71 Olomouc, Czech Republic

**Keywords:** plant development, adventitious root, genetic control, phytohormones, response to the environment

## Abstract

The spermatophyte root system is composed of a primary root that develops from an embryonically formed root meristem, and of different post-embryonic root types: lateral and adventitious roots. Adventitious roots, arising from the stem of the plants, are the main component of the mature root system of many plants. Their development can also be induced in response to adverse environmental conditions or stresses. Here, in this review, we report on the morphological and functional diversity of adventitious roots and their origin. The hormonal and molecular regulation of the constitutive and inducible adventitious root initiation and development is discussed. Recent data confirmed the crucial role of the auxin/cytokinin balance in adventitious rooting. Nevertheless, other hormones must be considered. At the genetic level, adventitious root formation integrates the transduction of external signals, as well as a core auxin-regulated developmental pathway that is shared with lateral root formation. The knowledge acquired from adventitious root development opens new perspectives to improve micropropagation by cutting in recalcitrant species, root system architecture of crops such as cereals, and to understand how plants adapted during evolution to the terrestrial environment by producing different post-embryonic root types.

## 1. What Are Adventitious Roots?

**Figure 1:**
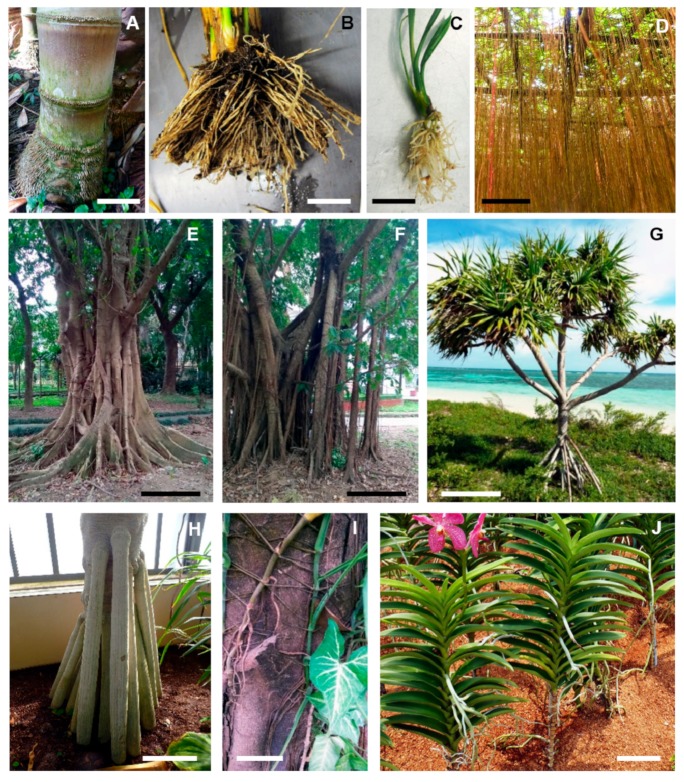
The illustration of the diversity of adventitious roots. (**A**) Bamboo stem with crown roots emerging at each node (scale bar: 15 cm), (**B**) fibrous root system of rice made by crown roots (scale bar: 3 cm), (**C**) fibrous root system of date palm tree seedling made by adventitious roots (scale bar: 1 cm), (**D**) hanging adventitious roots formed from *Bougainvillea* sp. (scale bar: 10 cm), (**E**) adventitious roots merging with the trunk of a *Ficus* sp. tree (scale bar: 50 cm), (**F**) pillar adventitious roots supporting branches in *Ficus* sp. (scale bar: 50 cm), (**G**) stilt roots in *Pandanus* sp. (scale bar: 50 cm), (**H**) adventitious roots establishing a protecting cage around the trunk in *Pandanus* sp. (scale bar: 1 m), (**I**) gripping adventitious roots and nourishing adventitious roots in a *Philodendron* sp. (scale bar: 5 cm), (**J**) aerial adventitious roots in an epiphyte orchid (scale bar: 5 cm), (Photographs A, B, C, D, G, H and J: M. Gonin, Université de Montpellier, France; photographs E, F and I: P. Gantet, Université de Montpellier, France).

The root system of spermatophytes is composed of a primary root that develops from the radicle formed during embryogenesis. Post-embryonically, the primary root can branch and form lateral roots. Post-embryonic roots can develop from stems, leaves or other vegetative non-root organs, either constitutively or in response to stress. Such roots are referred to as adventitious, a term that etymologically indicates their unusual and surprising origin (Figure 1) [1]. Adventitious roots (AR) are widely present in many dicot and monocot species in which they often differentiate from the stem. When AR develop constitutively from nodes, they are called nodal roots. Most monocots are characterized by a fibrous root system composed essentially of AR that constitutively develop from stem nodes (Figure 1A–C) [2,3]. In cereals, AR are named crown roots (CR). CR differentiate with a circular pattern all around the stem at each below ground stem node (Figure 1A) [2,4]. Brace roots constitute a category of AR that develops from the aerial nodes of maize-helping plant anchorage in soil [5]. Most spermatophyte species can develop AR constitutively and/or inductively in response to environmental signals, such as mechanical damage, flooding, biotic stress or in response to hormones during tissue culture [1,6,7]. The conservation of AR production capacity allows for a developmental plasticity that is beneficial to plants.

## 2. What Are the Roles of Adventitious Roots?

AR arose during the evolution of land plants because they provided the ability to adapt to different ecosystems. Together with lateral roots, AR play an important role in hydromineral uptake by increasing the volume of explored and exploited soil, and in plant anchorage necessary for the development of tall aerial vegetative apparatus [3]. AR increase the number of independent connections between the aerial plant body and the root system, conferring to the plant an increased capacity to tolerate pathogen or herbivore attacks leading to root damage [8]. In humid tropical forests, trees such as *Ficus* sp. produce many hanging AR that allow exploring and exploiting the aerial canopy where organic substrate-rich niches are frequent in cavities formed at tree branching sites (Figure 1D). AR can serve to consolidate the trunk by fusing with it or by developing buttresses (Figure 1E). For some woody species, AR can support the horizontal development of master branches by forming pillars (Figure 1F) [3]. Sometimes AR allow the plant to move towards better environmental conditions as observed for *Socratea exorrhiza*. In this case, AR are called stilt roots. Stilt roots develop from the trunk of palm trees under fallen limbs and obstacles to move out of the shadow, whereas at the opposite side of the trunk, the roots rot and die. The process is slow, but the tree can move with a velocity of approximately 1 m per year [9]. Stilt roots also increase plant height (Figure 1G) and the ability of a species to rapidly exploit light at the top of the canopy of tropical rainforests [10]. AR can also form a cage to protect the main trunk from herbivore attacks (Figure 1H) [3]. Semi-epiphytic plants such as philodendron can produce two types of AR from the nodes: the first type embraces the trunk of the host plant to ensure its anchorage, and the second type grows down, vertically along the trunk of the host plant to feed the epiphyte (Figure 1I). Epiphyte orchids form aerial AR that directly catch rain and dew water (Figure 1J) [3].

In conclusion, because of their ontological and morphological diversity, AR, in addition to improving the nutritive capacity and stability of plants, play a major role in the adaptation of plants to various environments.

## 3. Where Are Adventitious Roots Formed?

AR always develop from cells neighbouring vascular tissues [7]. In Arabidopsis, the plant model for dicots, AR initiate from hypocotyl pericycle-like cells [11], from the vascular tissues of the secondary growth in de-rooted hypocotyls or from the vascular tissues of stem cuttings [12,13]. Usually, AR formation begins with stem pericycle-like cell asymmetric divisions [14,15]. Interestingly, AR initiated from the pericycle-like layer via the formation of a microcallus-like structure [16]. Indeed, during in vitro micropropagation from the whole leaf of Arabidopsis, the vascular tissues of the petiole, including xylem and pericycle-like cells, first undergo a massive cell division to form a microcallus, which undergoes a second step of reprogramming specifying the AR founder cell afterwards [16]. It was recently reported that in Arabidopsis, the initiation of AR from different tissues requires the formation of a callus prior to the differentiation of the roots [17]. Such callus expresses the *WUSCHEL related homeobox 5 (AtWOX5)*, which plays a key role in root stem cell niche specification and maintenance, suggesting that callogenesis and rhizogenesis share, in part, genetic elements [18]. This is also supported by the fact that callogenesis and rhizogenesis cannot be induced in the *aberrant lateral root formation 4 (alf4)* mutant, impaired in lateral root formation, suggesting that both AR and induced calli derive from the xylem and the stem pericycle-like cells [16,18,19,20]. In the future, *AFL4* orthologs could be used in other species to determine whether the cell layers from which AR initiate possess a pericycle-like identity or not.

**Figure 2:**
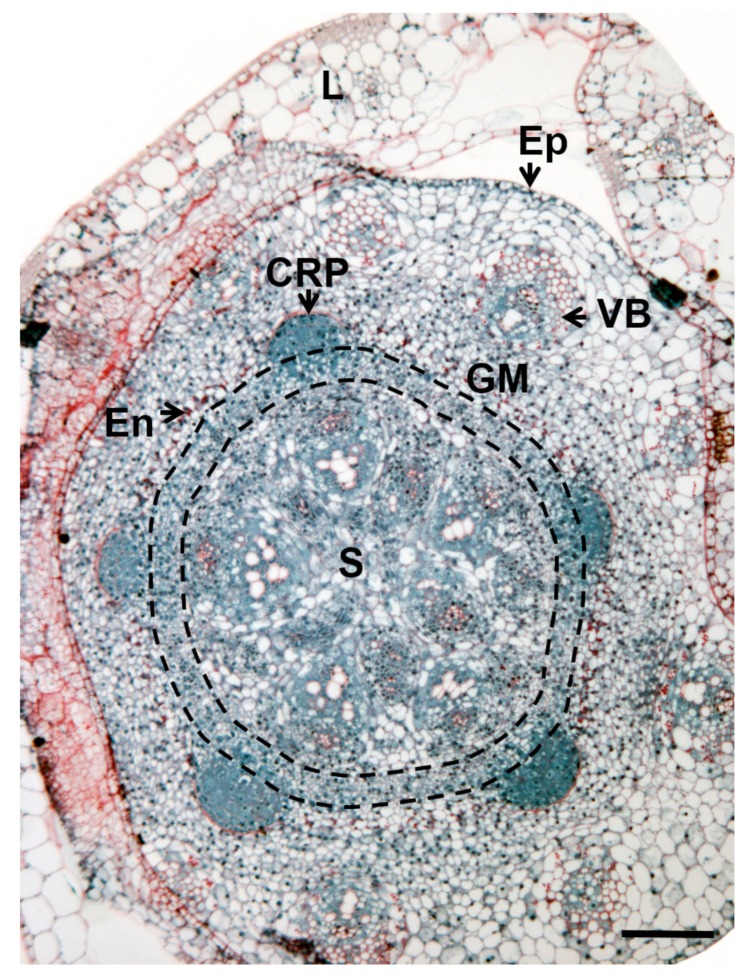
The rice stem transversal section showing the crown root initiation area. Staining: periodic acid-Schiff and naphtol-blue-black. CRP: crown root primordium; Ep: epidermis; En: endodermis; GM: ground meristem; L: leaf; S: stele; VB: vascular bundle. The black dotted lines indicate the separation of the ground meristem. Scale bar, 50 µm.

In rice, the plant model for monocots, CR differentiate from a cell layer called the ground meristem that has been assimilated to a stem pericycle-like tissue [3,21,22,23]. The ground meristem is located between the central zone that contains the vascular tissues and the starch sheath, constituted of cells rich in starch granules. This starch sheath corresponds to the shoot endodermis [17,24] (Figure 2).

In woody species, AR primordia also initiate from cells located between the vascular bundles and the starch sheath layer [25]. In cuttings, AR can develop from stem pericycle-like tissue adjacent to the vascular bundles or from the calli that differentiate after wounding [26,27]. In woody species such as cottonwood, carnation, poplar or apple tree, AR emerge from cells close to the vascular system of the stem [28,29,30,31]. For example, in young cottonwood hypocotyls, AR founder cells are adjacent to the vascular bundles and associated with poles of primary vascular tissues. In older seedlings, the same localisation is observed and the peripheral cells to the vascular bundles appear to retain intrinsic competence to form AR [28].

Besides their pericycle-like cell origin, AR can also initiate in Arabidopsis, petunia or woody species from phloem or xylem parenchyma cells, young secondary phloem cells, cambium cells close to the phloem cells, cotyledon parenchyma cells, leaf, and procambium [11,18,32,33].

Altogether, regardless of the species, tissue or organ, AR initiation occurs from pericycle-like cells and can associate vascular tissues and starch sheath, suggesting a common origin of AR formation in monocots and dicots, including woody species. Interestingly, callogenesis and rhizogenesis share genetic characteristics. This process resembles the two-step mechanism observed during hormone-induced organogenesis, previously proposed as an indirect pathway for AR induction in difficult-to-root woody species [34].

## 4. When Are the Adventitious Roots Produced?

**Figure 3:**
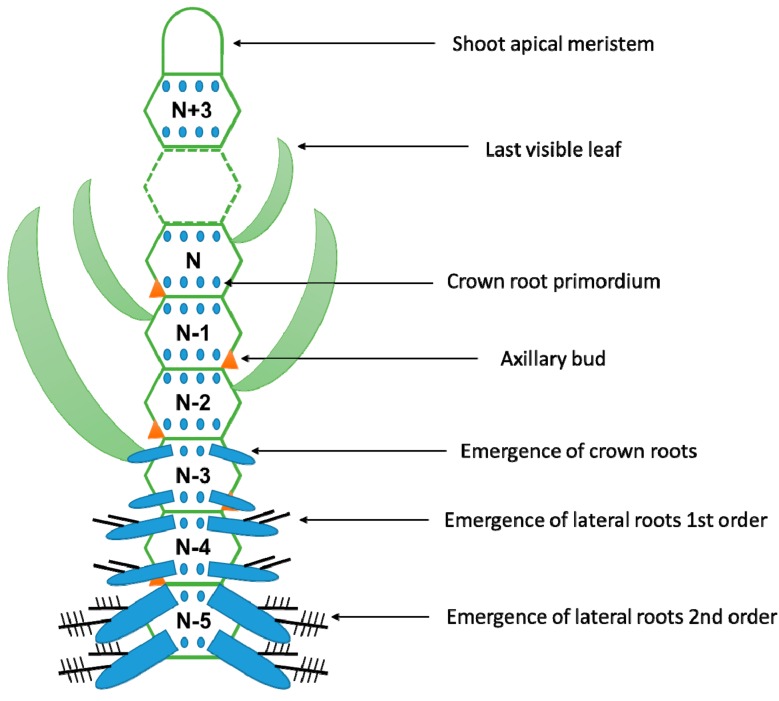
The phytomer and root-shoot synchronization in rice. The phytomer is made up of an axillary bud, a leaf, and a node (N). In rice, two rings of crown-root initia are present at both sides of the node (adapted from Reference [2]).

As already mentioned, AR are constitutively produced by several species. In many of them, at least one AR is systematically produced at each node. In rice, CR emergence from the stem is synchronized with the phyllochron. The phyllochron is the time interval between the initiations of two successive phytomers. A phytomer is a developmental unit composed of a leaf, a node, an axillary bud, and two rings of CR initiated at each side of the node. When a visible leaf emerges from the Nth phytomer, a ring of CR emerges from the stem of the N^−3^ phytomer (Figure 3). Thereby, the mature rice AR system develops acropetally and is composed of CR of different ages [2,35].

Beside constitutive AR, plants can produce AR in response to the environment: in response to anoxia stress provoked by flooding [36,37], to dark-light transitions such as a dark storage period condition for cuttings or light control of auxin homeostasis [38,39,40], to stem cutting [12,21,39,41] and to hormones [17,42]. Table 1 provides a non-exhaustive list of species for which adventitious roots are described in the present review.

The response to wounding or cuttings allows vegetative propagation that is used for cloning and multiplication of many forestry and horticultural plants [58]. In this context, AR formation involves responses to two primary stimuli: wounding at the cutting site and the isolation of the cut part from the whole plant resources. An extra stimulus such as exogenous hormone application is sometimes required to induce AR formation at the cutting site (see hereafter). In Arabidopsis, AR develop from de-rooted hypocotyls of old plants in which secondary growth has initiated or from the stem by cuttings [12]. This ability to develop new roots when the initial root has been removed is a strategy to ensure the plant’s survival upon root herbivore attack. During layering, another method of propagation, AR can develop from stems that remain attached to the parental plant and that are in contact with a medium favourable to rooting [59]. Reproduction by layering is a beneficial factor when seeds are not viable or growing in adverse conditions [60]. For example, vegetative reproduction through layering is a common strategy for woody species’ survival in the harsh climatic conditions of the alpine or northern timberline [60]. In this case, AR formation is stimulated by wet raw humus and by the mechanical effects of the high snow layer pressing branches down to the soil. Layering is a vegetative multiplication strategy used by many plant species. In Arabidopsis, AR can be induced by dark–light treatments [43]. In response to *Agrobacterium rhizogenes* infection, responsible for the “hairy roots” disease at the site of infection, AR can be induced from any vegetative part of the plant through the transfer of the bacterial *ROLB* gene into the plant host cell, modifying the endogenous hormonal balance in the transformed cells. These AR are agravitropic, highly branched, fast-growing and synthesise compounds called opines that feed the bacteria [3]. This system has been extensively used as the roots of many species are able to produce secondary metabolites of therapeutic interest. The ability to form AR varies between species and depends also on the type of organs and tissues.

Flooding is a severe abiotic stress that strongly affects plant physiology, while significantly reducing gas exchanges between the plants and their environment. This oxygen deprivation can go from the total absence of oxygen (anoxia) to low oxygen (hypoxia). A common adaptation to flooding is the formation of AR with aerenchym. In some species, such as tomato, AR are formed de novo upon flooding; in other species, such as rice or bittersweet, the primordia are constitutively preformed in the stem and their emergence is induced by flooding [55,58,61]. While deep AR is a beneficial trait for water uptakes, shallow-depth AR help to avoid hypoxic soil layers. In Arabidopsis, inhibition of growth of the main root system and the promotion of AR elongation under hypoxia restrict the root system in upper soil layers where the oxygen shortage may last for shorter time periods [36]. This is also supported by the observation of tobacco plants producing more AR in the hypoxia condition, but with reduced maximum length [37]. AR can contribute to flooding adaptation by taking up oxygen [38,55]. A greater AR porosity and stable root aquaporin expression suggest that AR are likely the key factors to maintaining high rates of root hydraulic conductance and shoot gas exchange in flooding conditions [37].

## 5. What Resource Availabilities Influence the Formation of Adventitious Roots?

**Figure 4:**
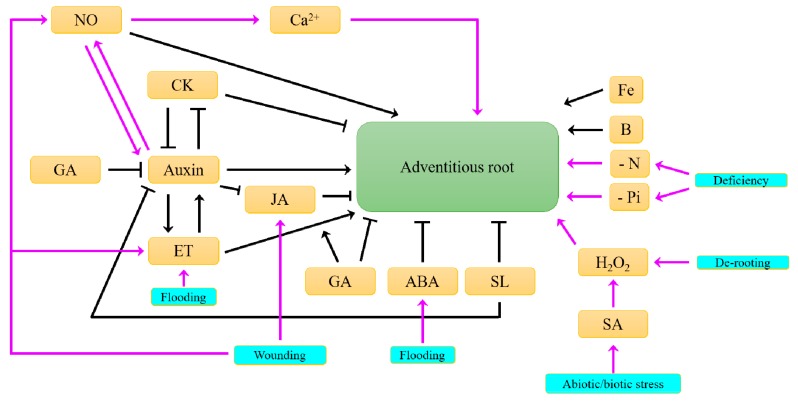
The signals acting on adventitious root formation. The squared text and black arrows describe the regulation of endogenous signals on the constitutive regulation of adventitious root formation. The blue squared text and purple arrows describe the action of external signals that induce the adventitious root formation. ABA: abscisic acid, Ca^2+^: calcium; B: boron; CK: cytokinin; ET: ethylene; GA: gibberellin; H2O2: hydrogen peroxide; Fe: iron; JA: jasmonic acid; N: nitrogen; NO: nitric oxide; Pi: phosphorus; SA: salicylic acid; SL: strigolactone.

The availability of water and macronutrients also regulates AR development [39] (Figure 4). Under nitrogen or water limitation, CR initiation and early development are inhibited in maize, but a foraging behaviour of the existing CR is favoured towards deeper soil horizons for water and nitrogen acquisition [40,41]. Based on these observations, Lynch in 2013 proposed a maize ideotype based on experimental and hypothetical data to optimise water and nitrogen acquisition by maize root systems [8]. Deep roots with high branching, different growth angles and large diameters for the primary root and small diameters for the seminal roots characterised this ideotype that optimised soil exploration [8]. Moreover, in high-input agroecosystems, parsimonious root phenotypes may benefit water and nitrogen uptake. In this context, the maize root system should have only a few AR, reduced density of lateral roots and reduced growth responsiveness to local resource availability [42]. In rice and bean, phosphorus starvation slightly stimulates the primary root elongation, inhibits lateral roots development, and increases AR initiation and elongation [57,62]. Mineral nutrition, i.e., Ca^2+^, nitrogen, zinc, phosphorus, iron and manganese, influences AR development of cuttings and the survival capacity in adverse conditions [26]. In petunia cuttings, iron promotes AR formation and acts locally by promoting cell division in the meristematic cells of AR primordia [52]. An exogenous supply of CaCl_2_ increases AR formation in Arabidopsis, most likely via the activation of mitogen-activated protein kinases. Similarly, the application of Ca^2+^ chelators and a Ca^2+^ channel blocker inhibits AR formation [63]. The micronutrient boric acid is required for AR formation in sunflower hypocotyl cuttings [26].

Whereas phosphorus is a crucial macronutrient for plant growth and development, its availability in soils is well below the concentration required for proper plant development. Moreover, in most soils, the phosphorus available to plants is present primarily in topsoil horizons, decreasing with soil depth. Its fast-chemical immobilization makes fertilization inefficient to overcome phosphorus deficiency [64,65,66]. In phosphorus-efficient genotypes of common bean (*Phaseolus vulgaris* L.), the limitation of phosphorus stimulates adventitious rooting, allowing topsoil foraging [64]. Nevertheless, the shallowing of the root system upon phosphorus deficiency is a trade-off for agriculture as it is often associated with a lower tolerance to drought.

The understanding of how external signals are perceived, conveyed, and interpreted to drive root adaptive developmental processes are still poorly known and should be further studied.

## 6. What Hormonal Signals Control the Formation of Adventitious Roots? 

Hormones are important regulators of the plant response, transducing external signals into physiological behaviour. Our current knowledge of the signals interplay involved in constitutive or inducible AR formation is summarized in Figure 4.

Auxin is the major growth-promoting hormone for AR initiation. Auxin such as indole-3-acetic acid (IAA) promotes AR formation, and auxin homeostasis is associated with different developmental steps of the rooting process [26]. In various plant species, higher auxin concentrations are required more for AR formation during the early steps of development than during the later steps [25]. Early steps in AR formation involve the IAA gradient and accumulation in specific cell types, via polar auxin transport (PAT) and local auxin biosynthesis, conjugation and degradation [7]. IAA biosynthesis has been identified to be essential for AR formation and PAT plays a key role in IAA distribution and gradient establishment [7,32]. For instance, IAA overproduction in Arabidopsis drives spontaneous AR formation in the hypocotyl [67]. Auxin accumulation is crucial to induce the priming of the founder cells that will later give rise to AR primordia. For instance, defects in PAT inhibit or modify founder cell priming and cells division involved in the AR formation, leading to a reduction of AR formation [27]. Following CR initiation in rice, auxin accumulation increases in root primordia and declines in epidermal cells above primordia suggesting that auxin signalling is involved in the coordinate processes of epidermal cell death and AR development through the surrounding tissues [68]. Local auxin maximum controlled by PAT through auxin efflux carriers has a key role in AR architecture in rice [68]. Many mutants in auxin biosynthesis, perception or signalling have been characterized and their functional study has highlighted some of the molecular mechanisms involved in AR formation downstream of the auxin signalling pathway [7].

Cytokinins (CK) and auxin act antagonistically in a wide array of physiological responses, including AR formation. In fact, an increase in auxin and the concomitant CK depletion are necessary for adventitious rooting in cuttings and often enough for AR in easy-to-root species [44]. Exogenous auxin treatment negatively affects CK biosynthesis in nodal stems of mung bean and carnation cuttings leading to AR [69,70]. In cuttings treated with benzyl adenine and indole-3-butyric acid (IBA), the same cell layers divide but form calli instead of roots [71]. The trade-off between cell differentiation and division controlling root meristem size and root growth is regulated by the antagonistic actions between CK and auxin in Arabidopsis and rice [72,73,74].

In response to wounding, wounding-related compounds, such as ethylene, jasmonate and nitric oxide, in association with auxin can promote cell division leading to AR formation [25,44,75].

In Arabidopsis, tomato and rice, flooding conditions induce the formation of AR via the local accumulation of ethylene that inhibits auxin transport, leading to an auxin accumulation that, in turn, induces ethylene production and increases the process of AR formation [26,53,58]. It has been proposed that ethylene regulates AR formation by controlling the localization and transcription of several auxin-efflux and -influx carriers, notably while regulating the PIN-FORMED (PIN) protein expression pattern [76]. In Arabidopsis, ethylene increases the expression of *PIN3* and *PIN7*, resulting in the modification of auxin transport [77,78]. Due to changes in PIN localization, ethylene regulates the distribution of AR initiation sites on the apical part of the hypocotyl, which shows more AR than the basal part in response to ethylene [78,79]. Moreover, ethylene regulates adaptive trait under prolonged flooding [80].

Ethylene may also represent the first warning signal indicating hypoxia and anoxia provoked by submergence [81]. In Arabidopsis seedlings, APETALA/ETHYLENE RESPONSE FACTOR (AP2/ERF) VII transcription factors control the establishment of the root system architecture in response to ethylene. While in Arabidopsis growth of AR is enhanced by hypoxia to explored topsoil layers and avoid an oxygen deficiency, growth of the main root system is inhibited [36]. These data are supported by previous studies where oxygen limitations have been shown to reorganize the plant root architecture [53,82]. Moreover, the ERF-VII transcription factors involved in the regulation of anaerobic genes also repress several genes by interfering with the auxin signalling pathways. The down regulation of key auxin-induced genes could contribute to proper root branching, allowing the establishment of an efficient root system [83].

In mung bean, ethylene promotes AR formation by increasing cells responsiveness to auxin [84]. Contrarily, gibberellins inhibit AR formation by stabilising PIN proteins and then perturbing the establishment of the PAT necessary for AR initiation [54,85]. In a recent study, gibberellins seem to be involved in the hypoxia response and AR development inhibition in barley [86]. Under osmotic stress, ethylene and calcium (Ca^2+^) are involved in promoting AR development [45].

Jasmonate and auxin crosstalk controls the AR initiation via AUXIN RESPONSIVE FACTOR (ARF) proteins and the *Gretchen Hagen3* (*GH3)* gene family, which encodes enzymes responsible for the conjugation of various amino acids to auxin and jasmonate, leading to their activation, inactivation, or degradation (see hereafter) [87,88,89]. ARF and GH3 regulate the level of jasmonoyl isoleucine, the active form of jasmonate which, in turn, negatively regulates adventitious rooting through the activation of the CORONATINE INSENSITIVE1 (COI1) signalling pathway [90]. In addition, in petunia cuttings, it was proposed that the peak of auxin produced in response to wounding decreases the jasmonate accumulation, favouring AR formation [7]. Nitric oxide, another wounding-related compound, is involved in the conversion of IBA into IAA that induces AR formation [91]. Nitric oxide increases auxin binding to its receptor, TRANSPORT INHIBITOR RESPONSE 1/AUXIN SIGNALING F-BOX (TIR1/AFB) and, consequently, the activation of auxin-mediated gene expression [92]. In rice, nitric oxide is essential for the CR primordia initiation: a reduction in endogenous nitric oxide inhibits the CR primordia initiation and decreases CR number [93]. In cucumber, nitric oxide acts downstream of auxin via cyclic guanosine monophosphate (cGMP)-dependent and cGMP-independent pathways to regulate AR formation [46,47]. The cGMP-dependent pathway involves the induction of calcium-dependent protein kinases, whereas the cGMP-independent pathway involves mitogen-activated protein kinases signalling cascade that regulates cell division in a (Ca^2+^)-dependent way [48,49,50]. After wounding, nitric oxide promotes Ca^2+^ accumulation into the cytosol that activates both calcium-dependent protein kinases and mitogen-activated protein kinases, leading to cell division involved in AR development [33,44,94]. Involvement of Ca^2+^ in AR development in response to nitric oxide has been also observed in cucumber submitted to osmotic stress [95]. A study on osmotic stresses during AR development in mung bean cuttings shows that nitric oxide interacts with auxin in response to abiotic stresses during AR development [96]. Further studies should be performed to assess the role of Ca^2+^ in the mechanism and to unveil the conserved processes among several species.

Salicylic acid that is involved in various biotic and abiotic stress responses promotes AR formation [51]. Salicylic acid significantly increases AR in a dose-dependent manner via the accumulation of free hydrogen peroxide (H_2_O_2_). In mung bean, H_2_O_2_ stimulates the expression of many genes involved in AR development [97]. A high level of H_2_O_2_ is a signal involved in AR formation after cucumber de-rooting, and the inhibition of H_2_O_2_ suppresses AR formation [97]. In this regard, the inhibition of the activity of the peroxidase, the enzyme responsible for the degradation of H_2_O_2_, has been correlated with AR formation in a dose-dependent manner, particularly during the early steps of initiation. The depletion of peroxidase activity is related to the auxin-dependent inhibition of the peroxidase isozyme de novo synthesis [98].

In conclusion, phytohormone crosstalk, with auxin as a hub, occurs in response to environmental signals in order to tightly and spatiotemporally control the AR development. Nevertheless, the major part of the cited studies focused on the characterization of one specific hormonal pathway during AR formation.

## 7. What Are the Genetic Determinants Controlling Constitutive Adventitious Root Formation in Rice?

**Figure 5:**
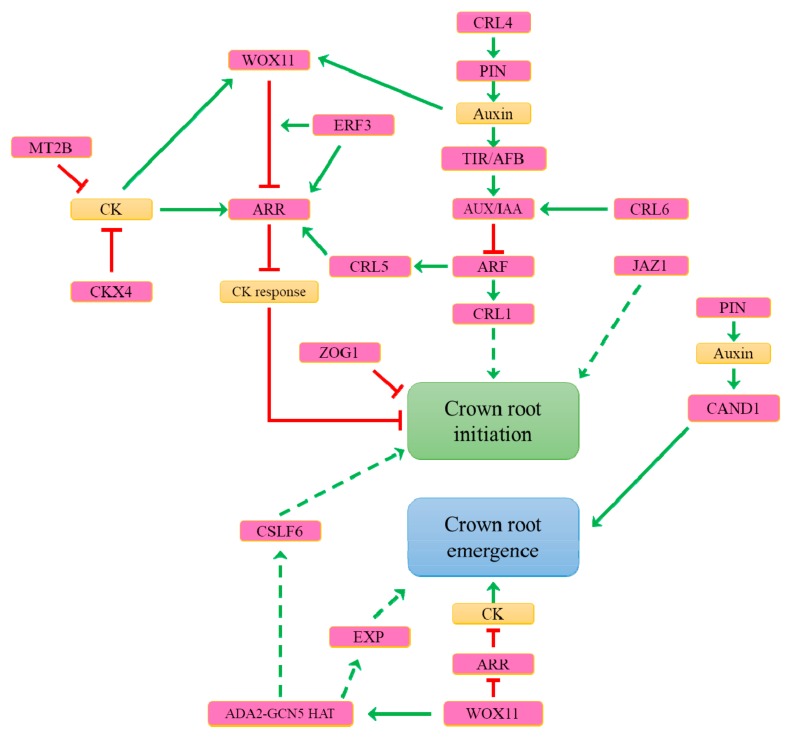
The molecular mechanisms of constitutive crown-root formation and emergence in rice. See the place of the two most important hormones in this process: auxin and cytokinins (CK) indicated in orange boxes. The green lines show positive interactions and the red lines show negative interactions. The dotted lines represent hypothetical interactions. Abbreviations: ARF: AUXIN RESPONSIVE FACTOR; AUX/IAA: AUXIN/INDOLE ACETIC ACID; CSLF6: CELLULOSE-LIKE SYNTHASE FAMILY 6; CRL1: CROWN ROOT LESS 1; CRL4: CROWN ROOT LESS 4; CRL5: CROWN ROOT LESS 5; CRL6: CROWN ROOT LESS 6; CAND1: CULLIN-ASSOCIATED AND NEDDYLATION-DISSOCIATED 1; CK: cytokinin; ARR: cytokinin-responsive regulator; CKX4: cytokinin oxidase 4; ERF3: ETHYLENE RESPONSE FACTOR 3; EXP: EXPENSIN; HAT: ADA2-GCN5 histone acetyltransferase; IAA: INDOLE ACETIC ACID; JAZ1: JASMONATE-ZIM DOMAIN 1; MT2b: METALLOTHIONEIN2b; PIN: PIN-FORMED; TIR1/AFB: TRANSPORT INHIBITOR RESPONSE 1/AUXIN SIGNALING F-BOX; WOX11: WUSCHEL-Related Homeobox 11; ZOG1: ZEATIN O-GLUCOSYLTRANSFERASE.

As already mentioned, cereals constitutively produce AR. Based on the analysis of rice and maize mutants impaired in CR development, genes involved in polar auxin transport and auxin signalling pathways were identified as major genetic components controlling CR formation [2,4,22,99,100,101,102] (Figure 5). In rice, CR development involves genes encoding TIR1/AFB auxin receptor and AUXIN/INDOLE ACETIC ACID (AUX/IAA), ARF, and LATERAL ORGAN BOUNDARIES DOMAIN (LBD) transcription factors and related regulatory proteins [101,103]. Rice mutants affected in the auxin biosynthesis pathway or PAT such as *tryptophan aminotransferase of arabidopsis* (*taa1)* [102] or *crown root less 4 (crl4)*/*Osgnom1* [104,105], respectively, exhibit few CR.

A phylogenetic analysis of the ASYMMETRIC LEAVES 2-like (ASL)/LBD family showed that the rice genes *OsLBD3-2* and *CROWN ROOT LESS1 (CRL1)* are very close to the maize *ROOTLESS CONCERNING CROWN AND SEMINAL ROOTS (RTCS)* that is involved in CR and brace roots formation [100,106,107]. *CRL1* induction is lost in plants overexpressing a non-degradable modified form of OsIAA3 [103]. *CRL6*, a gene encoding a chromatin remodelling factor involved in CR formation, stimulates the expression of several *OsIAA* genes [108]. In rice, 277 genes are positively regulated after the *CRL1* inducible overexpression in the *crl1* mutant background [109]. Among these genes, key genes are involved in cell proliferation, hormone homeostasis and root meristem patterning such as *QUIESCENT-CENTER SPECIFIC HOMEOBOX (QHB),* the rice orthologue of *AtWOX5.* A quarter of these genes have no homologs with Arabidopsis genes with a role in lateral roots initiation, suggesting that specific mechanisms regulate CR formation. *In situ* hybridisation localized *CRL1* expression pattern with some of these genes in CR initia and primordia [109].

In rice, several genes controlling CR formation are involved in auxin and CK signalling crosstalk. First, *CRL5,* which encodes an AP2/ERF family transcription factor, is induced by auxin, likely via OsARF1, and positively regulates the *cytokinin-responsive regulator* gene *OsRR1,* an inhibitor of the CK pathway [110]. The additive phenotype observed in the *crl1/crl5* double mutant suggests that *CRL5* acts through an auxin-related pathway distinct from *CRL1* to control CR initiation. Another transcription factor, the *WUSCHEL-Related Homeobox 11 (OsWOX11)* gene, is induced by auxin and CK, affecting auxin- and cytokinin-responsive gene expression. In the auxin biosynthesis *yucca* and *taa1* mutants, *OsWOX11* is downregulated and its overexpression can partially restore the formation of CR that is impaired in both mutants [102]. *OsWOX11,* required for auxin-dependent CR development [102,111], interacts with the rice AP2/ERF protein ERF3 and OsRR2 in different ways over CR primordia development. *ERF3* regulates *OsRR2* expression and is involved in CR initiation, whereas when *OsWOX11* is expressed after the CR primordia formation, the ERF3/OsWOX11 interaction likely represses *OsRR2* during CR elongation [112]. The pathways controlled by *CRL5* and *OsWOX11* do not interact, which suggests different regulatory pathways of CR development integrating auxins and CK [110,112]. Further studies on *OsWOX11* demonstrate its key role in CR development. *OsWOX11* regulates approximately 700 genes involved in root development, stress response, hormone signalling, and redox metabolism. Of them, 34% contain at least one *WOX cis*-regulatory motif *TTAATGG/C* [111,113,114]. The ChIP-PCR analysis from the roots reveals that *OsWOX11* binds the promoter of *OsLOB16*, homologous to *CRL1* [114]. Interestingly, in Arabidopsis, *AtWOX11*, orthologous to *OsWOX11*, also binds the *AtLBD16* promoter, promoting the root primordium identity during Arabidopsis adventitious rooting in vitro [115]. Other genes are involved in the control of AR formation in relation to CK. For example, when *METALLOTHIONEIN2b (OsMT2b)* is downregulated by RNA interference, the CK concentration increases and the number of CR decreases, whereas *OsMT2b*-overexpressing lines are characterised by an increase in CR and a lower amount of endogenous CK [116]. Similarly, the cytokinin oxidase OsCKX4 mediates the CR development in rice via the irreversible degradation of CK [74], whereas ZEATIN O-GLUCOSYLTRANSFERASE (OscZOG1), responsible for the glucosylation of *cis*-zeatin, negatively regulates the CR development formation [117].

The characterisation of rice, maize and Arabidopsis mutants altered in the CR or lateral root formation shows that a core regulatory network involving auxin-signalling pathways and LBD transcription factors is conserved during CR and lateral roots initiation, independently from the stem or root origin of the newly form post-embryonic root, respectively [101].

Taking into consideration these recent advances in the deciphering of genetic elements controlling AR, this confirms that auxin and CK pathways represent the main genetic control of AR formation. Nevertheless, as discussed previously, the role of other hormones should not be underestimated. Therefore, further genetic and functional genetic studies will undoubtedly help to clarify the hormonal crosstalk involved in the regulation of AR formation.

## 8. What Are the Main Determinants Involved in Inducible Adventitious Root Formation?

**Figure 6:**
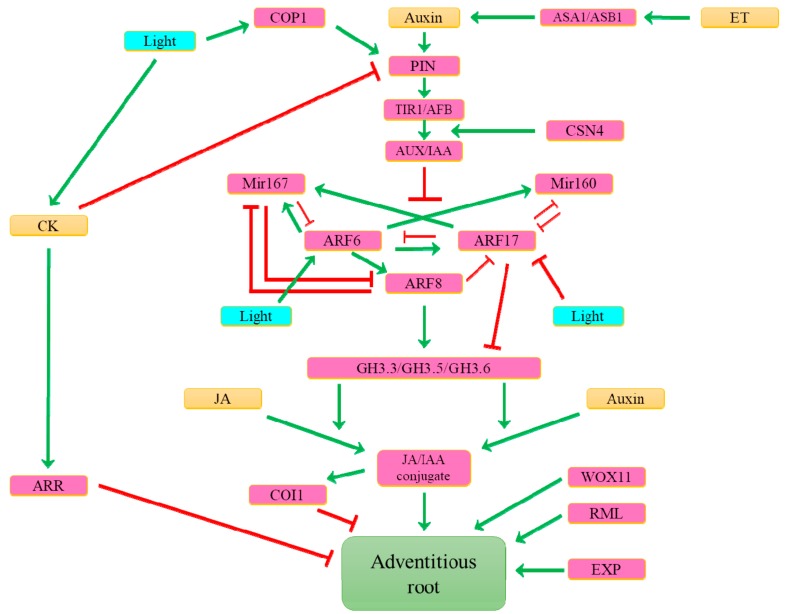
The integration of genes (pink), hormones (orange) and environment (blue) to control the inducible adventitious rooting in Arabidopsis. The green lines show positive interactions and the red lines show negative interactions. Abbreviations:; ASA1: ANTHRANILATE SYNTHASE ALPHA 1; ASB1: ANTHRANILATE SYNTHASE BETA; ARF: AUXIN RESPONSIVE FACTOR; AUX/IAA: AUXIN/INDOLE ACETIC ACID; COP1: CONSTITUTIVE PHOTOMORPHOGENIC 1; CSN4: COP9 signalosome subunit 4; COI1: CORONATINE INSENSITIVE 1; CK: cytokinin; ARR: cytokinin-responsive regulator; ET: ethylene; EXP: EXPENSIN; GH3: Gretchen Hagen3; IAA: INDOLE ACETIC ACID; JA: jasmonate; miR: microRNA, PIN: PIN-FORMED; RML: ROOT MERISTEMLESS; TIR1/AFB: TRANSPORT INHIBITOR RESPONSE 1/AUXIN SIGNALING F-BOX; WOX11: WUSCHEL-Related Homeobox 11.

In cuttings, endogenous auxin accumulation at the basal cut site is enough for AR formation induction in easy-to-root species, whereas exogenous auxin treatments are essential to stimulate AR formation from difficult-to-root species [44]. Comparison between easy- and difficult-to-root lines of *Eucalyptus globulus* showed that in cuttings, the endogenous content of auxin was two-fold higher in the easy-rooting line, confirming the importance of the hormone in AR induction [118]. Moreover, the Arabidopsis auxin-overproducing mutants *SUPERROOT* (*sur1* and *sur2*) and transgenic lines overexpressing the *YUCCA* gene involved in auxin biosynthesis spontaneously produce AR from hypocotyls because of auxin overproduction [33]. In Arabidopsis de-rooted hypocotyls, AR formation is stimulated by light. This AR formation involves three ARFs (ARF6, ARF8 and ARF17) that are regulated by specific miRNAs (miR167 and miR160) at the posttranscriptional level and that regulate the expression of three *GH3* genes [43,90]. The complexity of the interaction is emphasized by the integration of the regulatory loops between the *ARF* and *miRNA* genes in the regulation of *GH3* genes and AR formation [90]. Interestingly, in stem cuttings of black walnut *(Juglans nigra L.)*, the same three ARFs are involved in AR formation [119]. This observation suggests that these three ARFs may be key elements of AR formation in response to auxin conserved across diverse species. Other studies on woody species also suggest the key role of the GH3 gene family in AR formation by modulating auxin homeostasis. Indeed, the difference between easy-to-root and difficult-to-root genotypes is attributed to the variation in the concentration of the inactive form of auxin conjugates. The inhibition of auxin conjugation increases the rooting capacity, confirming that the auxin inactivation by conjugation via GH3 is a key step in AR formation [33]. Light-induced AR initiation in Arabidopsis requires PAT [11,44]. For example, in response to light, the photomorphogenesis repressor *RING E3 ubiquitin ligase CONSTITUTIVE PHOTOMORPHOGENIC1* (*COP1*) controls the transcription of the auxin efflux carrier gene *PIN1* that tightly regulates the distribution of auxin across the plant and influences, in particular, AR initiation [120]. In the Arabidopsis *sur2-1* double mutant, the AR are suppressed by the mutation of COP9 signalosome subunit 4 (CSN4), controlling the activity of *CULLIN-RING E3 ubiquitin ligases (CREL)*. CREL ubiquitinated proteins are then targeted to proteasomal degradation. The defect in AR formation in the *sur2-1/csn4* mutant is the result of the partially impaired degradation of the TIR1/AFB-auxin receptor, which alters auxin signalling and the ARF6/ARF8 regulatory module [121]. Additionally, in Arabidopsis, ARF and GH3 control the inducible adventitious rooting by modulating the free content of IAA and jasmonate, the latter being an inhibitor of adventitious rooting. Indeed, the hypocotyl of the *gh3.3-1, gh3.5-2* and *gh3.6-1* triple mutant, developing few AR compared to the wild-type, is characterized by a two-fold increase of endogenous free active jasmonate [90]. During AR initiation, auxin induces the expression of *GH3* genes, whose corresponding enzyme might conjugate jasmonate with the Asp, Met or Trp, leading to its inactivation and release of its inhibitory effect on AR initiation [90]. Despite data suggesting crosstalk between IAA and jasmonate pathways, information concerning jasmonate’s mode of action in the specific step of AR development is still limited. Like auxin, gibberellins are also involved in the accumulation of *GH3* transcripts and stimulate AR formation [54], underlying the complex hormonal regulation controlling AR formation. The complexity of this hormonal control is also evidenced by the upregulation by the ethylene signalling pathway of two genes encoding enzymes involved in auxin biosynthesis (ASA1/WEI2 and ASB1/WEI7) [122]. In cuttings grown in a light condition without exogenous auxin, the absence of rooting could be partially explained by the light-induced CK accumulation in leaves [123]. The complexity of the signalling network is reinforced by the analysis of Arabidopsis mutants. For example, mutants affected in gibberellins biosynthesis and signalling, auxin homeostasis, and xylem differentiation suggest their role in the AR formation. Interestingly, these mutants displayed the same AR pattern from the leaf culture or hypocotyls, suggesting common regulatory pathways essential for de novo organ formation in various organs [124]. The genetic determinants involved in inducible AR formation and described here are summarised in Figure 6.

The study of rice growing in adverse conditions, such as drought stress, allowed the identification of other genes involved in hormone-regulated CR development. For example, the overexpression of the *JASMONATE-ZIM DOMAIN1* (*OsJAZ1)/EXTRA GLUME2 (EG2)*, induces the production of more, larger and longer CR. *OsJAZ1* was retrieved from a Quantitative Trait Locus for drought resistance, suggesting for the first time that the JAZ protein belongs to the determinants of the root traits both under normal and drought stress conditions in rice [125].

In pine cuttings, the genes of the *expansin* (*EXP*) family, also involved in post-embryonic root formation in Arabidopsis, are among the early auxin-induced genes activated before cell divisions and AR formation [126]. *AtWOX11*, an Arabidopsis ortholog of *OsWOX11,* promotes the root primordium identity during Arabidopsis in vitro adventitious rooting [115]. In poplar, two paralogs of *OsWOX11* have been identified. The constitutive overexpression of *PtWOX11* enhanced the rate of rooting and increased the numbers of AR per cutting [127]. This suggests that *WOX11* likely controls a well-conserved mechanism for AR formation in monocots and dicots [111,127].

In conclusion, at the molecular level, the understanding of AR development in model species suggests the conservation of some genetic determinants of AR formation. The availability of genome editing tools such as CRISPR-Cas^9^ guided RNA, and the genome sequences of diverse taxa will help to validate the conservation of these mechanisms in non-model plants.

## 9. What Are the Mechanisms Controlling Adventitious Root Emergence?

In rice, after initiation, CR develop inside the stem and stop growing. Later, CR growth restarts, allowing their emergence from the stem epidermis. This mechanism might be involved in the synchronization of CR emergence and shoot development, as mentioned before. The emergence of CR corresponds to the development of CR primordia through the stem tissues, through cells that have suffered programmed cell death. The polar auxin transport controls AR elongation and regulates the growth direction of emerging AR [68].

In addition to this constitutive development, CR emergence can be induced by external signals, such as flooding or exogenous ethylene but not by gibberellins, auxin, or CK treatment [3]. In CR primordia, ethylene is required for the induction of cell cycle regulatory genes, and CR emergence is promoted by the induction of epidermal cell death at the emerging site [3]. The localised programmed cell death of stem epidermal layers is necessary for AR emergence from the stem [56]. In flooded rice, ethylene and gibberellins have a synergistic effect that promotes AR emergence by inducing the programmed cell death of the stem epidermal layers, whereas abscisic acid negatively controls the AR emergence [56]. In addition to its role in AR formation, auxin is also involved in CR emergence in rice. The rice *cullin-associated and neddylation-dissociated 1* (*Oscand1)* mutants show normal CR primordia formation but no CR emergence [128]. *OsCAND1* regulates CR growth through the stem by the control of the cell cycle at the transition G2/M in CR meristem initial cells. In Arabidopsis, the *root meristemless* (*rml*) mutant is impaired in lateral roots and AR emergence and elongation, suggesting the role of *RML* in post-embryonic root elongation [129]. *RML* encodes a γ-glutamylcysteine synthetase involved in glutathione biosynthesis [129]. The inhibition of glutathione biosynthesis causes cell cycle arrest and defects in auxin signalling that affect the QC maintenance and, therefore, the growth and cell divisions in the root [129]. Some genes involved in AR formation are also involved in AR emergence. This is well illustrated by the recent deepening of the function of *OsWOX11*. After initiation, *OsWOX11* is expressed in emerging CR in division regions of the root meristem where, in association with OsERF2, it activates CK signalling by downregulating *OsRR2* [112]. *OsRR2* is expressed in CR primordia and its repression by OsWOX11/OsERF2 may be necessary to promote the emergence of CR [111]. OsWOX11 recruits the ADA2-GCN5 histone acetyltransferase that is required for auxin signalling gene expression in the Arabidopsis root meristem [130] and that is highly expressed in the root meristem and essential for cell division and growth. Then, the OsWOX11/ADA2-GCN5 histone acetyltransferase complex activates downstream target genes in the CR meristem for energy metabolism, cell wall biosynthesis, and hormone response that are necessary for root meristem cell proliferation during CR development [131]. Some of the potential targets of OsWOX11 are involved in the rooting process. For example, OsCSLF6, a putative cellulose-like synthase family member, is required for rice root development in response to phosphate [131,132]. Rice expansins, such as OsEXPA8, OsEXPA17, and OsEXPB5, also modulate root emergence [133,134,135]. Genetic determinants involved in AR emergence are summarised in Figure 5.

## 10. What Represses Adventitious Root Formation?

Many hormones play a role in the negative control of AR development to maintain the trade-off between carbon cost and development (Figure 4, Figure 5 and Figure 6). As previously mentioned, CK are antagonistic to auxin, suppressing AR formation in many species, including Arabidopsis, rice, and poplar [136,137,138]. In Arabidopsis, zeatin riboside is the primary suppressor of adventitious rooting originating from hypocotyls [139]. It has been demonstrated that in this context, CK inhibit cell differentiation in the region between the root elongation zone and the cell division zone but have no effect on cell proliferation in the root meristem [72]. Moreover, AtARR1, AtARR10 and AtARR12 directly bind to the promoter of the *YUC4* gene to block its activation, repressing auxin biosynthesis, again underlying how CK regulates auxin homeostasis to inhibit AR formation [140]. The inhibitory effect of CK on poplar adventitious rooting results partially from the action of PtRR13 (a cytokinin type-B response regulator) on the PAT by stimulating the expression of a gene encoding the auxin efflux pump *PLEIOTROPICDRUGRESISTANCE TRANSPORTER9* (*PtPDR9*) [138]. Exogenous CK treatment also acts directly on founder cells to inhibit adventitious rooting in eucalyptus [141].

JA inhibits AR initiation in etiolated Arabidopsis hypocotyls through the COI1 signalling pathway. As already mentioned, COI1 is controlled by complex crosstalk between the miRNA, ARF and GH3 proteins that regulate jasmonate conjugation and the subsequent effect of jasmonate on AR development [90]. ARF17 negatively controls the AR initiation by repressing the expression of the *ARF6, GH3.3, GH3.5,* and *GH3.6* genes that are involved in the auxin and jasmonate homeostasis regulation [90]. In stem cuttings of black walnut (*Juglans nigra* L.), ARF17 seems to have a conserved function; nevertheless, further study would be required to confirm the conserved mechanism across diverse taxa [119].

Abscisic acid is also a negative regulator of AR development in tomatoes [1]. The abscisic acid-deficient tomato *flacca* and *notabilis* mutants produce additional AR. In the *notabilis* mutant, the phenotype of the WT could be rescued by expressing the *9-cis-epoxycarotenoid dioxygenase1* (*SpNCED1)* gene involved in abscisic acid biosynthesis [142]. In rice, exogenously applied abscisic acid decreases the AR formation by up to 50% [56].

Gibberellins also have a negative effect on AR formation. In the poplar mutants *gibberellins insensitive* (*gai*) and *repressor of GA1-like*1 (*rgl1*), affected in gibberellins perception and responses to gibberellins, AR formation was unaffected [143]. In tomato, the gibberellins constitutive mutant *procera* had a low capacity to produce AR in a root-inducing medium [85]. Gibberellins treatment inhibits the CR formation in rice, whereas a rice mutant deficient in gibberellins biosynthesis develops additional AR [144]. Surprisingly, an opposite response was observed in flooded rice in which gibberellins promoted the initiation of CR in the presence of ethylene [56].

Strigolactones repress the AR development by inhibiting the first divisions of founder cells in different species, including Arabidopsis, tomato, pea, and maize [145,146]. They likely act in interaction with ethylene, CK, and auxin. For example, in Arabidopsis and pea, the basipetal auxin transport and auxin accumulation in the rooting zone are negatively regulated by strigolactones [146]. In cuttings, the negative effect of strigolactones on the AR formation is blocked due to the absence of the major source of these phytohormones, the roots. Strigolactones seem to promote CR formation in rice by modulating auxin transport [147]. The species-specific of strigolactones effect on adventitious rooting raises questions that remain to be answered.

## 11. What Are the Main Perspectives of Research on Adventitious Rooting?

Although our knowledge on the mechanisms of AR formation and their regulation by environmental signals has increased in the last past few years, it remains limited compared to information related to mechanisms involved in primary root and lateral root formation and development. In this regard, several questions must be addressed. For example, what are the common or different developmental pathways involved in AR, primary root and lateral root development? Why are AR constitutively formed in some species but inducible in others? Would a species with a taproot system benefit from evolving a fibrous system and vice versa?

Studies of species such as maize and rice provide clues concerning AR regulatory networks and are useful to complete our understanding of AR constitutive development. Genetic and molecular studies related to AR development allow the initiation of a model of AR formation regulation in rice [103]. This knowledge begins to have an application in breeding strategies aiming to shape the root system architecture in relation to specific environmental constraints [148] and might also be useful to facilitate the vegetative propagation of woody and horticultural species of interest for which AR formation is the main limiting step. The differences in AR production capacity among species are not understood yet. The analysis of ageing and DNA methylation levels of cuttings as limiting factors of AR formation should be explored [149,150]. Comparison of genotypes and species with differing abilities to produce AR and the identification of a Quantitative Trait Locus associated with AR development capacity should help to understand AR development mechanisms. The *ROLB* gene from *A. rhizogenes* has been described as the most effective gene in promoting root formation, and the use of this gene in genetic transformation to improve the AR of some recalcitrant plants [151], such as apple [152] and pear trees [153], is one example of using knowledge to overcome the limits to rooting.

Root adaptive development in response to drought that limits the AR formation in the topsoil while promoting already formed AR development in deep soil horizons in maize [154,155] may have a genetic basis. The use of 3D imaging technologies such as X-ray computed tomography and PET-MRI [156,157] to investigate root systems at a global level in natural conditions could help identify genes involved in AR adaptive developmental responses. For example, in rice, the overexpression of *OsERF3*, which plays a role in AR development, increased grain yield under severe drought conditions but not under normal conditions [158]. Most of the published reviews on the role of auxin and the crosstalk between different hormones during AR development make a parallel with the data available for lateral root development. An effort to clarify the regulatory networks involved in lateral roots and AR development may help to better understand the post-embryonic rhizogenesis process and its evolution. The analysis of fossil plant structure indicates that roots first appeared during land plant evolution from points along the stem, being therefore essentially of shoot-borne origin. Branches with a specific rooting function were first observed among relatives of the lycopods during the Early Devonian (416–398 Ma). The earliest seed plants, also known as seed ferns, had roots developing throughout the aerial systems, frequently borne along the stems and sometimes organised to most likely play a role in buttressing. The origins and early evolution of roots went hand in hand with the development of shoots. Specialised rooting systems were necessary for the evolution of increasingly larger and more sophisticated plants capable of exploiting a broad range of habitats [159]. Dicot and monocot plants have a distinct ability to form secondary tissues. In this regard, the abundance of constitutively formed AR in monocots could be a compensation trait to cope with the absence of capacity to produce secondary vascular tissues, which increase transport capacities from soil to aerial part of the plant in aged dicot plants. Thus, understanding the mechanisms involved in AR development could help to better understand the origin of the root organs and the basis of their evolution to the current complex and diverse root systems observed in spermatophytes.

## Figures and Tables

**Table 1:** A non-exhaustive list of species discussed in this review for which adventitious roots have been described to occur constitutively and/or in an inducible manner. The source of induction is indicated. n.d.: not determined. Numbers indicate the reference describing the process.

Species		Constitutive	Inducible
**Dicots**	Arabidopsis	no	mechanical damage [12] dark/light [11,43,44] flooding [26,36] in vitro culture [16,18]
	Cucumber	n.d.	salt [45] mechanical damage [46,47,48,49,50]
	Epiphyte	yes [3]	n.d.
	Ficus	yes [3]	n.d.
	Mung bean	n.d.	mechanical damage [36,51] biotic stress [51]
	Petunia	n.d.	mechanical damage [6,7,32,52]
	Sunflower	n.d.	mechanical damage [26]
	Tobacco	n.d.	flooding [37]
	Tomato	n.d.	flooding [53]
**Monocots**	Barley	yes [1]	flooding [54]
	Maize	yes [1,5]	n.d.
	Rice	yes [2,3]	flooding [38,55,56], Pi starvation [57]
	*Socratea exorrhiza*	yes	mechanical damage [9]
**Woody species**		yes	mechanical damage [26,27,28] in vitro culture [26,27,28]
